# SOLARIS project: a portable 3D-printed bioaerosol sampler for environmental bacterial collection

**DOI:** 10.1098/rsos.240364

**Published:** 2025-02-05

**Authors:** Pedro Henrique Dobroes Fonseca, Filipe Miguel Borgas Henriques Duarte, Frederico Silva de Sousa Alves, Jose Alberto de Jesus Borges, Susana Isabel Pinheiro Cardoso, Vania Cristina Henriques Silverio, Wilson David Talhao Antunes

**Affiliations:** ^1^ INESC MN, Lisboa 1000-029, Portugal; ^2^ Department of Bioengineering, Instituto Superior Técnico, Universidade de Lisboa, Lisboa 1049-001, Portugal; ^3^ Optimal Defence, Santarém 2005-256, Portugal; ^4^ Infectious Diseases Department, Instituto Nacional de Saúde Doutor Ricardo Jorge, IP (INSA), Lisbon 1649-016, Portugal; ^5^ Centro de Investigação, Desenvolvimento e Inovação da Academia Militar (CINAMIL), Instituto Universitário Militar, Lisboa, Portugal; ^6^ Department of Physics, Instituto Superior Técnico, Universidade de Lisboa, Lisboa 1049-001, Portugal; ^7^ Unidade Militar Laboratorial de Defesa Biológica e Química (UMLDBQ), Exército Português, Lisboa 1849-012, Portugal

**Keywords:** aerosols, bioaerosols, bioaerosol sampler, biosurveillance, biomonitoring, One Health

## Abstract

Bioaerosols, a subset of aerosols released from the biosphere, can carry pathogens, and include particles with diameters from nanometres to a few micrometres. They can remain suspended indoors and travel significant distances. Bioaerosol studies play a vital role in public health, as bioaerosols are an effective route for human and animal pathogen transmission, especially in animal production and handling facilities, which are considered hotspots for the emergence of zoonotic pathogens. The ‘One Health‘ approach, which interconnects human, animal and environmental health, underscores the need for robust biomonitoring and biosurveillance systems. We introduce the SOLARIS project, a novel bioaerosol sampler manufactured through three-dimensional printing with a biocompatible material. Our sampler is compact, portable and uses a liquid collection medium, increasing bioefficiency. Our sampler’s laboratory testing demonstrated the successful separation of viable *Escherichia coli* bacteria from artificially generated bioaerosols. Collected samples were found suitable for downstream analysis methods such as culturing, mass spectrometry, molecular detection and electron microscopy. A field trial at a swine facility was performed, in which *Clostridioides difficile* spores were successfully collected from bioaerosols and identified using microbiological and molecular methods, reinforcing our sampler’s utility and emphasizing the significance of incorporating aerosol samples in research studies within the One Health approach.

## 1. Background

Aerosols are particles suspended in the air or gas, ranging from nanometres (nm) to a few micrometres (µm) in diameter [1]. Bioaerosols are a subset of aerosols released from the biosphere that can carry pathogenic microorganisms [2]. Studies on animal farms have collected bacterial pathogens from bioaerosol particles with diameters smaller than 3 up to 20 µm and larger [3–6]. Bioaerosols and droplets from human and animal activities (e.g. breathing, coughing, sneezing) can remain suspended for varying durations depending on several factors, especially particle diameter. Furthermore, particles below 5 µm can remain suspended indefinitely indoors. Recent studies show that in indoor settings with controlled airflow (5−20 cm s^−1^), 5–10 µm bioaerosol particles settle farther than 6 m from the source and can travel 15−60 m. Low relative humidity and high ambient temperature negatively impact pathogen viability but positively affect travel distances by evaporating fluids and reducing particle sizes [7]. Bioaerosols with diameters from 5 to 2.5 µm, able to carry bacterial pathogens, can reach the pulmonary region of the respiratory system. This finding is critical for human and animal health, as this allows pathogens to bypass immune defences, stealthily gaining access to tissues and provoking severe illness [1,2].

Bioaerosol sampling can be passive or active. Passive sampling involves particle settling by gravity on a substrate, while active sampling relies on flow and particle inertia, and includes mechanical components. Active bioaerosol samplers consist of an inlet, sample transport system, a particle size selector, a collection medium and a calibrated airflow [8]. Using a liquid collection medium has been seen to improve bioefficiency by reducing sample desiccation [9,10]. Active bioaerosol samplers have different sizes and technologies, such as cyclones, impactors, impingers and filter sampling devices. Flow rate ranges from 2 to 800 l min^−1^, depending on the sampler type. The bioaerosol sampler choice considers the target microorganisms, sampling environment and device cost [11–13].

Cyclone-type samplers utilize centrifugal force to separate particles from the airflow based on their size, density and speed. However, collection losses may occur due to evaporation, re-aerosolization of collected material and liquid carryover [14,15]. Nonetheless, larger cyclone samplers have successfully captured bacteria, spores and viruses in indoor and outdoor environments [15]. As for impactor-type bioaerosol samplers, they use particle inertia for collection. Particle-laden air is jetted across a gap towards a collection medium positioned perpendicular to the nozzle outlet [16]. Here, the collection efficiency depends on particle diameter, density, nozzle diameter and air velocity of the jet airflow [17].

## 2. Study relevance and goals

Bioaerosol studies are essential for public health, as bioaerosols are established as a fundamental human and animal pathogen transmission route in various settings [18–30]. The ‘One Health’ approach links human, animal and environmental health in a collaborative and transdisciplinary manner. Animal production and handling facilities are hotspots for the emergence of zoonotic pathogens. The severe acute respiratory syndrome coronavirus 2 (SARS-CoV-2) is a zoonotic pathogen that reached pandemic potential, demonstrating the need for global surveillance strategies and tools for emergent pathogen monitoring.

In this study, we introduce the SOLARIS project, a novel 3D-printed bioaerosol sampler integrating a cyclonic cup and an impactor. To characterize the sampler’s collection efficiency and bioefficiency, aerosolization experiments were performed in a controlled environment, using a model bacterial organism, *Escherichia coli*. Different sample analysis methods such as bacterial culturing, mass spectrometry, nucleic acid extraction for molecular detection and electron microscopy were performed, for microbial viability assessment and validation of the bioaerosol sampler for downstream applications [11,31,32].

The SKC Biosampler^®^ aerosol sampler was selected as a benchmark in this study due to its extensive usage and characterization both in the laboratory setting and in the field, operating principle and characteristics comparable to our sampler and good collection performance in the 0.5–10 µm particle diameter range [33].

To establish a Technology Readiness Level (TRL) 6, a prototype system was tested in the field at a swine facility for the detection of *Clostridioides difficile*, a Gram-positive spore-forming anaerobic bacterium with clinical importance, found in the human and animal gut [34]. This highlights the role of bioaerosols in the transmission of pathogens and emphasizes the utility of incorporating aerosol samples in research studies within the One Health approach.

## 3. Methods

### 3.1. Computational fluid dynamics

We conducted system flow simulations using COMSOL Multiphysics 5.1 for a cyclone with four inlets, and a cyclone coupled to a virtual impactor with four inlets. The flow model was based on the Navier–Stokes equations, considering a Newtonian fluid (air) with constant density. Airflow was assumed to be stationary, and the walls were considered to have a no-slip condition. Air compressibility was obtained by defining the Mach number as the ratio of the local flow velocity to the sound velocity in the medium. We assigned an inlet boundary condition with a normal flow velocity equivalent to the volume flow rate determined by the suction pump, and assigned an outlet boundary condition with a pressure of 0 Pa. A computational fluid dynamics (CFD) module with physics-controlled meshing was used to solve fluid velocity and pressure in the system. By applying the simulation to the three-dimensional (3D) cyclone geometry, air velocity and pressure within the cyclone were calculated. Additionally, the trajectory of the biological entity (e.g. bacterial cell) was predicted using the particle tracing module, considering the biological entity as a spherical particle. Flow parameters and particle properties are presented in table 1.

**Table 1.** Flow parameters and particle properties used in the simulations.

property (unit)		value
ambient temperature (K)		293.15
ambient pressure (atm)		1
volume flow rate (m^3^ s^−1^)		1 × 10^–4^
air	density (kg m^−3^)	1.184
	dynamic viscosity (Pa s)	1.849 × 10^–5^
particle	diameter (µm)	1
	density (kg m^−3^)	1100
	total quantity on entry	840

We released 840 particles at the inlet(s) over 1 s, with 84 particles released every 0.1 s. Each particle was tracked from the injection to its destination (boundary or internal wall). Particle distribution at the inlets, outlet and other boundaries was monitored to assess the particle concentration ability. If a particle contacted an inner wall, it was considered to adhere to it. Flow computation in the device involved a two-phase gas–solid flow with one-way coupling. The cyclone was coupled to a virtual impactor to allow particle capture onto a liquid collection medium.

The computer simulation consisted of the following steps: defining geometry and materials using air with properties from table 1; setting inlet and outlet conditions (*Q*_input_ = 6 l min^−1^ (1 × 10^–4^ m^3^  s^−1^), *p*_output_ = 0 Pa) in the CFD module; specifying particle properties (density, diameter) in the particle tracing module; considering frictional force and gravity as the two forces acting on particles; constructing the mesh and performing calculations to support a stationary study (CFD module) determining the frictional force on particles, air velocity and pressure fields, followed by a particle trajectory study (particle tracing module); analysing simulation results to determine particle trapping efficiency. The analysis of simulated results supported the choice of design fabricated as explained in the next section.

### 3.2. Sampler design and fabrication

We fabricated most of the SOLARIS project bioaerosol sampler components using a dual extruder 3D printer (Da Vinci 1.0 Pro 3-in-1, XYZprinting, Taiwan). Polylactic acid (PLA) filament was the fusion polymer used for the parts. The precise layer-by-layer deposition technique of fused filament fabrication allowed efficient manufacture of the sampling housing, intake nozzle and internal flow path complex geometries. The air intake pump used to draw air through the sampler was powered by a compact 12 V, 2.0 Ah lithium-ion polymer battery mounted externally. The air pump streamed flows between 12 and 15 l min^−1^ (D2028, Airpon, China) and was assembled securely into the integrated sampling port using press-fit fittings. Initial flow rate testing with an analogue rotameter indicated that the controlled pump provided a target flow of approximately 6 l min^−1^ through the intake, during preliminary open-air sampling trials.

### 3.3. Controlled aerosolization tests

We aerosolized *E. coli* American Type Culture Collection (ATCC) 25922 strain (serotype O6, biotype 1) in a custom-built aerosolization chamber. The enclosure’s dimensions are 190 mm diameter, 200 mm height, 4.653 × 10^–1^ m^2^ internal surface area and 2.268 × 10^–2^ m^3^ volume.

#### 3.3.1. Bacterial suspension preparation

*Escherichia coli* ATCC 25922 taken from stock in bacterial freezing medium (VWR, Radnor, PA, USA) was grown for 24 h in 15 ml buffered peptone water (VWR) at 37°C, 1 ml transferred to a 1.5 ml Eppendorf tube, centrifuged at 5000 ×*g* for 10 min and the pellet was washed three times in 1 ml phosphate buffer saline (PBS). The suspension was diluted in PBS to an optical density at 600 nm (OD_600_) of 0.5 (≈1 × 10^8^ colony-forming units per millilitre (CFU ml^−1^)) using a spectrophotometer (Nanodrop ND-1000; ThermoFisher Scientific, Wilmington, NC, USA). Next, serial dilutions were done, plating −6 dilutions in plate count agar (PCA) culture medium (VWR), incubated at 37°C for 24 h and colonies counted to confirm the CFU ml^−1^ of the original culture. Finally, serial dilutions were performed by the previously described method to obtain fresh bacterial suspensions at 1 × 10^6^ CFU ml^−1^ for the aerosolization tests.

#### 3.3.2. Aerosolization efficiency tests

We calculated the microbial aerosolization efficiency of a nebulizer (NE-C28P, Omron Healthcare Europe BV, Hoofddorp, The Netherlands) capable of generating aerosol particles with a mass median aerodynamic diameter (MMAD) of 3 µm at a 0.5 ml min^−1^ aerosolization rate, using *E. coli*. Previous studies have demonstrated that particles of a similar size can carry bacteria in both laboratory and field settings [3–6].

A commercial bioaerosol sampler coupled to an air sampling pump (Biosampler^®^ and BioLite+pump, SKC Inc., PA, USA) was set at 12 l min^−1^ flow rate. On the other end, the aerosol sampler inlet was connected to the nebulizer reservoir outlet through 150 mm transparent silicone tube and a polypropylene (PP) adapter. The bioaerosol collection medium used was sterile PBS.

A 1 ml volume of a 10^6^ CFU ml^−1^ bacterial suspension in PBS buffer was aerosolized for 120 s and the generated bioaerosol was sampled for the next 120 s (24 l total) in triplicate. Next, the resulting 5 ml hydrosol samples were transferred to 15 ml high-clarity PP tubes. The nebulizer’s reservoir and the Biosampler^®^ were sterilized by autoclaving at 121°C for 15 min before the experiments. The Biosampler^®^ sample cup was decontaminated between triplicates with 0.05% sodium hypochlorite followed by 70% ethanol and drying at 160°C for 15 min. The resulting 5 ml hydrosol samples were centrifuged at 4600 × *g* for 6 min, resuspended in 2 ml PBS buffer and divided into two 1 ml aliquots. Serial dilutions were performed from −2 to −4 in 1 ml PBS and 100 µl aliquots were plated onto PCA culture medium. Cultures were incubated at 37°C for 24 h and colonies were manually counted. The remaining 1 ml aliquot of each experiment was used for quantitative polymerase chain reaction (qPCR) analysis.

Aerosolization efficiency ($\eta_{A}$) was calculated using the following equation:


$$\eta_{A}=\;\frac{W_{out}}{W_{in}}\times 100\;\left(\%\right),$$

where $W_{out}$ (CFU ml^−1^) is the number of microorganisms collected and counted in 1 ml of aerosolized microbial suspension and $W_{in}$ (CFU ml^−1^) represents the number of microorganisms counted in 1 ml of original microbial suspension.

#### 3.3.3. Bacterial deposition tests

We estimated bacterial losses by adhesion to the aerosolization chamber surfaces by placing eight polymethyl methacrylate (PMMA) squares (10 mm × 10 mm × 4 mm) on the chamber’s walls during a bioaerosol sampling test with our sampler and the Biosampler^®^, described in the next section. After the bioaerosol sampling test, each PMMA square was vortexed in 2 ml PBS. The PBS was then centrifuged, and the resulting pellet was resuspended in 100 µl PBS. Bacterial CFU were determined by plating onto PCA culture medium and colony counting, as previously described. Finally, total microbial loss due to deposition was estimated by multiplying the average deposition on each section of the chamber by the section’s total surface area and summing the values.

#### 3.3.4. Aerosol sampling tests

We aerosolized a 10^6^ CFU ml^−1^ *E. coli* suspension and sampled 24 l of air using our sampler for 240 s and the Biosampler^®^ for 120 s, to account for the difference in flow rate between the two bioaerosol samplers. The bioaerosol collection medium used was sterile PBS, 2 ml in our sampler and 5 ml in the Biosampler^®^. Sterilization and decontamination of the equipment were done as described previously. Resulting hydrosol samples were processed for colony counting and qPCR analysis. We corrected the number of aerosolized bacteria with the aerosolization efficiency value previously obtained. Bacterial losses through adhesion and deposition in the chamber walls were calculated by subtracting the CFU obtained in deposition tests from the number of aerosolized CFU.

Bioaerosol sampling efficiency ($\eta_{S}$) for each sampler was calculated according to Kesavan & Sagripanti [35], determined by the equation:


$$\eta_{S}=\;\frac{\left(\frac{N_{c}}{V_{s}}\right)}{C_{air}}\times 100\;\left(\%\right),$$

where $N_{c}$ is the number of microorganisms collected and counted (CFU) from a volume of air sampled $V_{s}$ (litres) and $C_{air}$ is the concentration of microorganisms in the air (CFU l^−1^).

### 3.4. qPCR tests

A calibration qPCR run was performed with known reference samples using DNA extracted from a bacterial suspension with an initial concentration of 5.7 × 10^8^ CFU ml^−1^ down to the −7 dilution and results were compared to colony counts by the *R*-squared method using Microsoft Excel software (*n* = 16). Additionally, qPCR Ct values before and after aerosol sampling using our sampler and the SKC Biosampler^®^ were compared using Wilcoxon signed-rank tests in IBM^®^ SPSS^®^ statistics software. To this end, DNA was extracted from three dilutions of samples before and after aerosolization with comparable colony counts, from each experiment with our sampler and SKC Biosampler^®^ (*n* = 18). Finally, a Spearman’s rank-order correlation was conducted to determine the relationship between qPCR Ct values obtained from samples collected using our sampler and the SKC Biosampler^®^. For this, DNA was extracted from the original samples and three dilutions with comparable colony counts, from each experiment with our sampler and SKC Biosampler^®^ (*n* = 24).

DNA extraction was performed from 1 ml of sample using a commercial kit (QIAamp^®^ DNA Mini Kit; QIAGEN, Venlo, The Netherlands). DNA was resuspended in 100 µl of pH 8.0 Tris–EDTA (TE) buffer. Colony counting and qPCR were done for each sample, and results were analysed in a spreadsheet software (Microsoft Excel^®^).

The qPCR reactions with 20 µl final volume were prepared using a commercial kit (LC Fast Start DNA Master SYBR Green kit; Roche, Basel, Switzerland) and primers for 16S bacterial genes (forward primer: 5′-AGA GTT TGC TCC TGG CTC AG-3′; reverse primer: 5′-GTA AGG TTC TTC GCG TTGC-3′) previously diluted to 20 µM. Reactions were set up according to the manufacturer’s instructions for a final volume of 20 µl, and performed in a real-time thermal cycler (LightCycler^®^ System; Roche). The qPCR protocol used was kindly provided by the Infectious Diseases Department of the Portuguese National Institute of Health Dr. Ricardo Jorge (Instituto Nacional de Saúde Doutor Ricardo Jorge, INSA, IP) and is available at https://dx.doi.org/10.17504/protocols.io.q26g7ped8gwz/v1.

### 3.5. Scanning electron microscopy

Scanning electron microscopy (SEM) images of *E. coli* samples collected with our sampler were compared with SEM images of *E. coli* taken directly from colonies in PCA culture medium to evaluate potential differences in bacterial cell morphology associated with bioaerosol sampling. An ultra-high-resolution field emission instrument (SU8010, Hitachi High-Tech, Tokyo, Japan), at an accelerating voltage of 1.5 kV, 8 mm working distance, in secondary electron mode was used. Two magnifications were employed: 2500× for a cellular organization overview and 10 000× for detailed bacterial morphology, and 10 randomly selected fields were visualized.

#### 3.5.1. Sample processing and imaging

*Escherichia coli* ATCC 25922 culture plate samples diluted to 10^4^ CFU ml^−1^ in 1 ml of PBS and hydrosol samples collected in 2 ml PBS with our sampler and 5 ml PBS with the Biosampler^®^ were processed using a filtration system comprising 250 ml funnels and 0.45 µm polyethersulfone (PES) filters (PALL^®^; Port Washington, NY, USA). Next, the samples immobilized in the PES filters were fixed in 2.5% glutaraldehyde and 1% formaldehyde in 0.1 M cacodylate buffer for 30 min, dehydrated in a series of ethanol of increasing concentration and placed in tertiary butyl alcohol. Samples were then mounted on metal microscope stubs, frozen at −20°C for 30 min and the tertiary butyl alcohol was evaporated under a vacuum. Afterwards, post-fixation with osmium tetroxide was performed and the samples were coated with an 8 nm gold layer using a sputter coater (model 108auto, Cressington Scientific Instruments Ltd, Watford, UK).

### 3.6. Bioaerosol sampling field trial

#### 3.6.1. Site description

We performed a bioaerosol sampling field trial at the swine maternity of the National Zootechnical Station of the Portuguese Agrarian and Veterinarian Research Center (Estação Zootécnica Nacional–Instituto Nacional de Investigação Agrária e Veterinária, INIAV, IP). The full sampling site description can be found in Alves *et al*. [34].

#### 3.6.2. Sample collection

We used our sampler and SKC Biosampler^®^ to sample 120 l of air (20 min at 6 l min^−1^ with our sampler and 10 min at 12 l min^−1^ with the Biosampler^®^) at 1.5 m from ground level. Collection of three samples was performed in the two maternity units, 2B and 3C, with natural ventilation through windows and doors. All samples were collected in PBS. Additionally, we sampled 50 healthy animals from both maternity units (piglets aged 0−3 weeks were assigned to unit 3C and 3- to 5-week-old piglets to unit 2B) to evaluate the animal population *C. difficile* carriage status. Five piglets and the corresponding sow were sampled for each litter. According to available biological material, we collected fresh droppings from the sows and rectal swabs from the piglets.

#### 3.6.3. Sample treatment, bacterial isolation and characterization

Field trial bioaerosol sample treatment, bacterial isolation and characterization were performed at the National Reference Laboratory for Gastrointestinal Infections from the Infectious Diseases Department of the Portuguese National Institute of Health (Instituto Nacional de Saúde Doutor Ricardo Jorge, INSA, IP)

From the three bioaerosol samples collected from each maternity unit, one was directly plated onto *C. difficile* agar medium (ChromID^®^, bioMérieux, Marcy l'Etoile, France) for quantitative analysis of *C. difficile* spore aerosolization. After ethanol shock and centrifugation of the PBS solution, pellets were inoculated onto the agar and the plates were incubated for 48 h at 37°C under anaerobic conditions.

The remaining two bioaerosol samples were enriched prior to inoculation. The enrichment protocol description and the protocol applied to the animal samples are described in Alves *et al*. [34]. We selected three presumptively positive samples based on colony morphology characteristics and subcultured them onto *Brucella* blood agar with hemin and vitamin K1 culture medium (BD BBLTM; Beckton-Dickinson, Franklin Lakes, USA) under anaerobic conditions for 24 h at 37°C.

After species confirmation by matrix-assisted laser desorption/ionization time-of-flight (MALDI-TOF) mass spectrometry (VITEK MS, bioMérieux), phenotypical and genotypical characterization, antimicrobial susceptibility testing, toxigenic profiling and ribotyping of the *C. difficile* isolates were performed as described by Alves *et al*. [34].

## 4. Results and discussion

### 4.1. Sampler design

The SOLARIS project bioaerosol sampler was manufactured using PLA, a biodegradable thermoplastic, by 3D printing. The sampler integrates two particle collection methods: a cyclonic cup and an impactor. In the cyclonic cup, air enters tangentially, creating a vortex that forces larger particles to the outer walls for collection. Smaller particles remain airborne and move to the impactor stage, where they are collected in a liquid medium as the air stream changes direction at the cyclonic cup exit. This dual-stage approach allows for efficient separation and collection of particles based on their size and inertia. and is a Many current samplers use either cyclonic or impaction principles separately. Unlike some existing impactors like the Andersen Cascade Impactor that use solid or semi-solid collection media, our sampler employs a liquid collection medium. This choice enhances bioefficiency and improves compatibility with various downstream applications, including culturing, mass spectrometry, nucleic acid extraction for molecular detection and electron microscopy, as demonstrated. The 6 l min^−1^ sampling flow rate of our sampler is an improvement over existing personal samplers, such as the SKC’s Institute of Occupational Medicine personal sampler, which operates at a lower flow rate. Our sampler’s higher flow rate allows increased sampling capacity while maintaining compact size.

CFD simulations showed that 1 µm particles were successfully transferred through the cyclonic stage and captured in the impactor stage. Bioaerosol sampling results demonstrated that our sampler effectively separated particles generated by the nebulizer with a MMAD of 3 µm, suggesting that its hybrid collection system approach allowed the collection of particles in the of size range of 1−3 µm.

Our bioaerosol sampler offers notable design advantages over existing systems, which are typically manufactured using traditional methods using materials like metal, plastic or glass. The SOLARIS project sampler can be provided as a do-it-yourself (DIY) kit. The main component of the sampler can be 3D printed locally using PLA, making our sampler a viable alternative for usage in developing nations, especially in rural and remote areas, or in emergency scenarios such as outbreaks or pandemics, when supply chain disruptions can occur. This also significantly reduces manufacturing and shipping costs. Additionally, the sampler can be decontaminated with chemical agents like hydrogen peroxide, chlorine solutions or glutaraldehyde, allowing multiple uses.

As the SOLARIS project sampler advances towards TRL 9, all technical specifications are expected to be validated. Upon commercial production in Europe, projected sale price would reflect the added value of professional assembly, quality assurance and distribution channels, but is expected to be significantly lower than the competition. Transition from a DIY kit to a commercially available product ensures that the system meets industry standards. This dual approach of a DIY kit and a commercially produced version maximizes accessibility and impact, ensuring that high-quality air sampling technology is available to both resource-limited settings and professional environments.

### 4.2. Computational fluid dynamics

**Figure 1.**
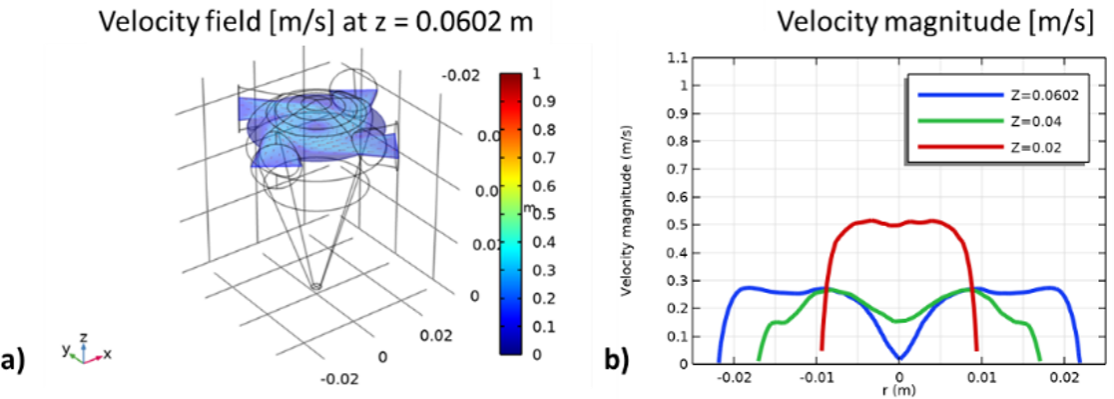
*(a*) Velocity field at plane *z* = 0.0602 m. (*b*) Velocity magnitude at planes *z* = 0.0602, *z* = 0.04 and *z* = 0.02 m for *y* = 0 m along the cyclone radius.

Figure 1 illustrates the distribution of velocity magnitude in the four-inlet cyclone. Figure 1*a* presents the velocity field at the *z* = 0.0602 m plane, where the velocity is highest near the centre of the inlets and decreases towards the cyclone centre. A homogeneous velocity distribution can be observed in the studied plane (figure 1*a*). Figure 1 compares the velocity modulus at different heights for *y* = 0 m along the cyclone radius. The velocity profiles indicate that the position of the inlets determines the maximum velocity at *z* = 0.02 m. The symmetry of the velocity field at this point affects the spiral motion around the centre of rotation (line *x* = *y* = 0 m and 0 ≤ *z* ≤ 0.069850 m) and, consequently, the cyclone’s ability to direct particles towards the exit. Velocity fluctuations at the cyclone bottom are visible in figure 1*b*, indicating a symmetrical velocity distribution. This design results in a velocity of approximately 0.5 m s^−1^ at the cyclone centre at *z* = 0.02 m, exceeding four times the inlet air velocity. The air approaches the centre and, due to the Coriolis force, experiences a leftward deflection, leading to clockwise rotation.

**Figure 2.**
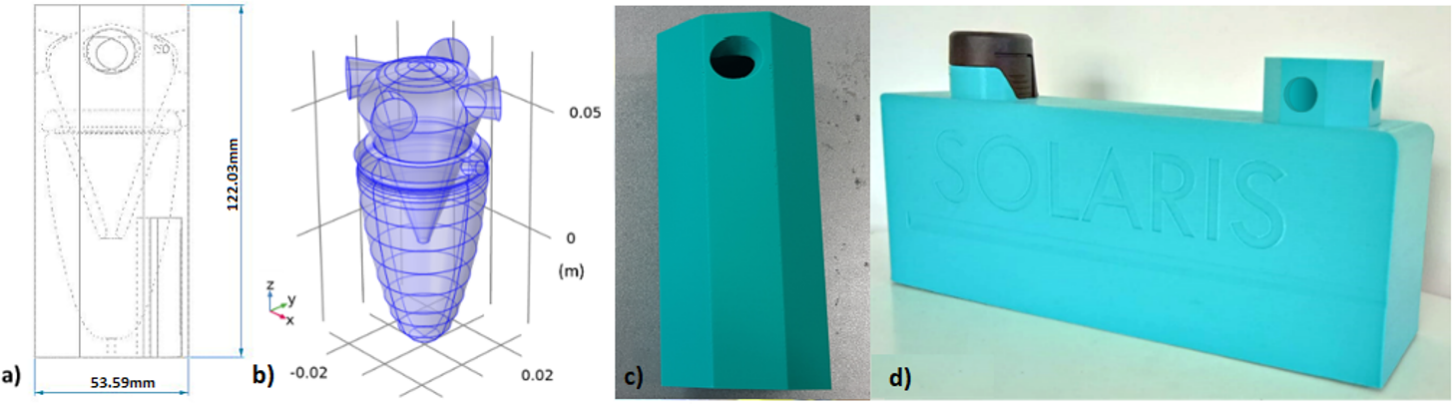
(*a*) Technical drawing with external dimensions in mm. (*b*) The 3D simulation structure of the virtual impactor coupled to the cyclone with four inputs. (*c*) The prototype fabricated in PLA by 3D printing. (*d*) The integrated system of the PLA 3D-printed prototype, with the suction pump on the left, 12 V batteries inside (not visible) and the sampler prototype on the right. The system is mounted in a holder and features the SOLARIS project logo.

Our sampler’s structure is visible in figure 2. Figure 2*a* shows a technical drawing featuring the outer dimensions in mm and the inner structure in transparency. Figure 2*b* shows the simulation structure of the virtual impactor coupled to the cyclone. Figure 2*c* shows the 3D-printed prototype in PLA. The printed version incorporates an additional outer layer. This layer was not included in the simulations, as it was found that it did not significantly affect particle capture efficiency. Figure 2*d* presents the 3D-printed prototype in a holder, together with the 12 V battery powering the suction pump (not visible).

**Figure 3.**
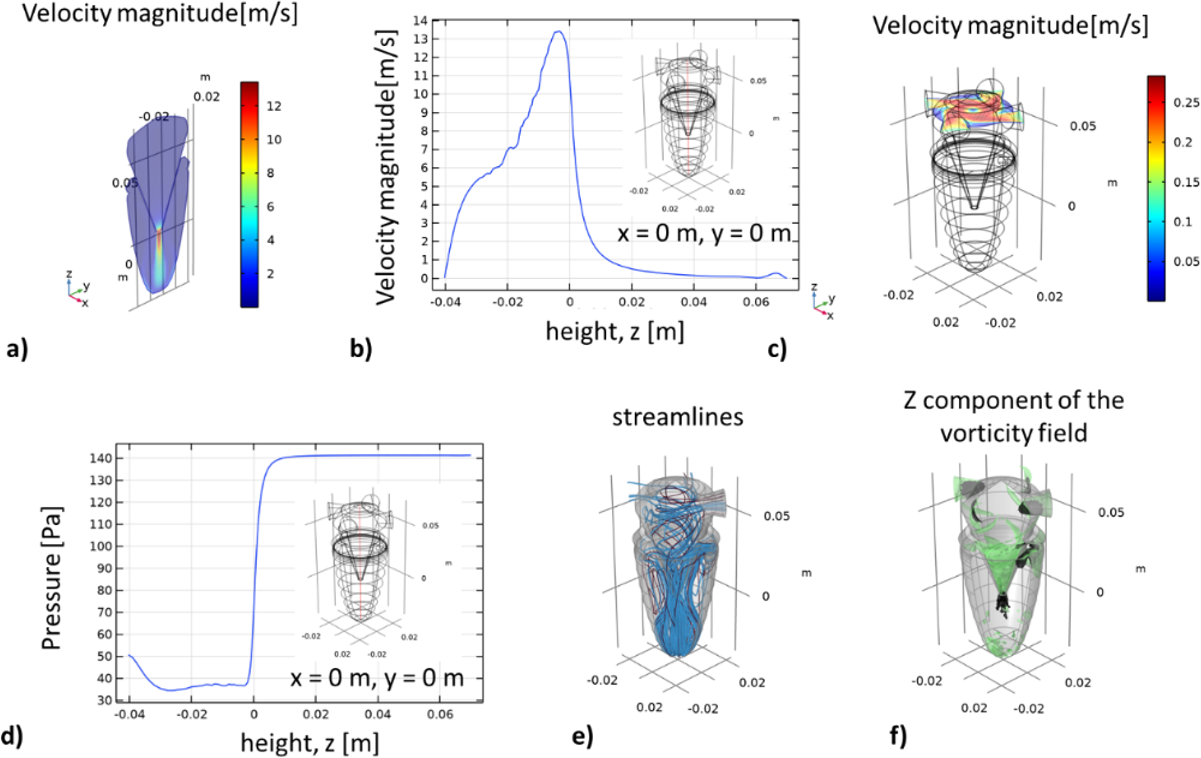
Air velocity, pressure and flow distribution in the cyclone–virtual impactor system. (*a*) Air velocity modulus in the *yz* plane at *x* = 0 m. (*b*) Distribution of air velocity modulus along the *z*-axis (bottom to top) at *x* = *y* = 0 m. (*c*) Air velocity modulus and flow distribution on the *xy* surface at *z* = 0.0602 m. (*d*) Pressure distribution along the *z*-axis (bottom to top) at *x* = *y* = 0 m. (*e*) Visualization of air current lines in the system. (*f*) Analysis of the *z* component of the vorticity field.

Figure 3 reveals that the bottom walls of the inlets are situated at *z* = 0.0602 m. Figure 3*a,b* indicates that the air velocity is approximately 0 m s^−1^, therefore within the prescribed 0.02 m ≤ *z* ≤ 0.069850 m range. Consequently, we hypothesize that gravity will draw particles below this point into the virtual impactor. Furthermore, we expect the particles to accelerate due to increased velocity near the cyclone exit (*z* = 0 m) and project towards the bottom of the virtual impactor. Figure 3*c* presents the velocity field at the *z* = 0.0602 mm plane. Like figure 1, the velocity modulus is highest at the centre of the inlets (*v*_max_~0.26 m s^−1^) and lowest (*v*_min_ = 0 m s^−1^) near the walls. The velocity is also lowest at the centre of the cyclone, as confirmed by the result shown in figure 3*b*. The pressure profile along the system’s centreline at *x* = 0 m, *y* = 0 m and −0.04031 < *z* < 0.06985 m is displayed in figure 3*d*. The proposed hypothesis is supported by the current lines that describe the airflow trajectory throughout the system (figure 3*e*). These curves are tangential to the flow’s velocity vectors. The impactor’s bottom, positioned at a 90° angle and facing the passage opening between the cyclone and the impactor, deflects the flow lines in the opposite direction. Under these conditions, particles encounter the impactor wall and adhere to it. Figure 3*f* illustrates the more intense *z* component of the vorticity field near the cyclone exit and the entry into the virtual impactor.

**Figure 4.**
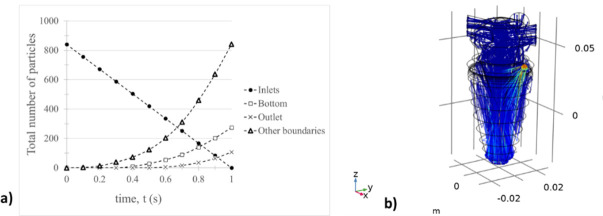
Particle analysis in the SOLARIS project aerosol sampler system. (*a*) Number of particles released at the inlets (circles) and their distribution: adhered to the bottom of the virtual impactor (squares), passed through the system outlet (crosses) and adhered to other boundaries (triangles). (*b*) Trajectories of particles in the system visualized using spherical particles with properties described in table 1.

The number of particles released in the particle tracing module (840) ensures independence of results according to Hari *et al*. [36]. Figure 4 illustrates the simulation results of particle trajectories and quantities. We observed that a fraction of particles adhered to the walls of the cyclone and the virtual impactor (other boundaries). Additionally, more particles were found to adhere to the bottom of the virtual impactor compared to those leaving the system, as indicated by the analysis of the airflow velocity field (figure 4*a*). Particle adhesion to the walls of the cyclone and virtual impactor, especially at the bottom, is observed, necessitating sample collection at the system’s base (figure 4*b*). This process results from coupling the cyclone with a virtual impactor. We verified that particle capture efficiency at the bottom of the virtual impactor is achieved through gravity-driven deposition and acceleration near the cyclone exit. Adding a liquid collection medium to the bottom of the impactor provides an optimal solution for particle collection.

From the computer simulation analysis, we conclude that the designed cyclone with four inlets effectively collects air samples from any direction. Simulation results revealed that the cyclone’s tangential and axial velocity components are significantly higher than the radial component. Moreover, positioning the four inlets enables a symmetric cyclone circulation, ensuring efficient particle transfer through the outlet and into the impactor. Finally, the 3D simulations also offered insights into the velocity and pressure profiles, confirming the expected particle motion within the system [37,38].

### 4.3. Controlled aerosolization tests

#### 4.3.1. Aerosolization efficiency tests

Following collection, the bacterial concentration was determined to be 7.8 × 10^5^ CFU ml^−1^ through colony counting. The aerosolization efficiency, calculated as previously described, was found to be 13.82%. Despite its relatively modest aerosolization efficiency, the system offers the advantages of affordability, compactness and ease of use, making it a practical option for our research needs.

#### 4.3.2. Bacterial deposition tests

In the first round of experiments using our sampler, the average bacterial CFU obtained from PMMA squares was 16 (*s* = 11; max = 36; min = 3), with an estimated 11% bacterial loss due to adhesion inside the aerosolization chamber. For the Biosampler^®^, the average bacterial CFU plate counted from PMMA squares was 10 (*s* = 10; max = 34; min = 1), with an estimated 5.83% bacterial loss due to adhesion inside the aerosolization chamber. Average plate counts obtained for the top, walls and base of the aerosolization chamber are in table 2.

**Table 2.** Bacterial deposition plate counts in CFU for the top, walls and base of the aerosolization chamber, obtained during the bioaerosol sampling tests performed with our sampler and the Biosampler^®^ and total estimated bacterial loss by adhesion inside the aerosolization chamber.

bioaerosol sampler		SOLARIS project	Biosampler^®^
		average plate counts (CFU)	
aerosolization chamber area	top	22	22
	walls	17	7
	base	8	5
estimated bacterial loss		11%	5.83%

The interaction between *E. coli* and PMMA surfaces involves non-specific forces initially, such as van der Waals, hydrophobic and electrostatic interactions. Some cells experience reversible adhesion, while others undergo irreversible adhesion facilitated by bacterial molecules like adhesins or fimbriae interacting with PMMA ligands [39,40]. The complexity of this process, along with the small sample size, can explain the variability in plate count values. PMMA surface treatment with 70% ethanol may also reduce *E. coli* adhesion by altering surface hydrophilicity, charge and chemistry.

#### 4.3.3. Bioaerosol sampling tests

Sampling efficiencies of 1.67% for our sampler and 4.01% for the Biosampler^®^ were obtained, as shown in table 3. Our experiments demonstrated that both devices’ sampling efficiencies are comparable, under the specific experimental conditions.

**Table 3.** Average plate counts obtained after the aerosolization tests with our sampler and with the Biosampler^®^ and calculated bioaerosol sampling efficiency for each bioaerosol sampler.

aerosol sampler	SOLARIS project			Biosampler^®^	
serial dilutions	−2		−3	−2	−3
average plate counts (CFU)	10		2	30	1
aerosol sampling efficiency	1.67%			4.01%	

**Table 4.** Experimentally obtained sampling efficiency value (NC) and corrected values using the average internal loss value minus 1 standard deviation ($\bar{x}-\sigma$ = 10.9%), average loss value ($\bar{x}$ = 21.1%) and average loss value plus 1 standard deviation ($\bar{x}+\sigma$ = 31.3%) according to Han & Mainelis [41].

aerosol collector	sampling efficiency value			
	NC	$\bar{x}-\sigma$	* $\bar{x}$ *	* $\bar{x}+\sigma$ *
SOLARIS project	1.67%	13.92%	25.38%	36.84%
Biosampler^®^	4.01%	15.59%	26.42%	37.25%

However, the Biosampler^®^ showed a significantly lower sampling efficiency value (4.01%) compared to the previously reported value of 25% by Zheng & Yao under similar experimental conditions [33]. Han and Mainelis investigated inherent and latent internal losses in liquid-based bioaerosol samplers, and reported an overall bacterial loss of 21.1 ± 10.2% for the SKC Biosampler^®^ when collecting 1 × 10^6^ CFU of *Bacillus subtilis* for 15 min at a 12.5 l min^−1^ flow rate. In table 4, we present the sampling efficiency values experimentally obtained and the sampling efficiency values corrected for overall internal bacterial losses according to Han & Mainelis [41].

Sampling efficiency values corrected for overall internal bacterial losses align more closely with those previously reported by Zheng & Yao [33]. We can, therefore, conclude that accounting for internal losses is crucial for accurate bioaerosol sampler performance assessment and that such analysis must be done in future studies using our sampler.

Negligible fluid loss by evaporation (50 µl PBS) from 5 ml collection volume over 2 min of sampling with the Biosampler^®^ at 12.5 l min^−1^ was estimated by Lin *et al*. [42]. Consequently, fluid loss by evaporation was assumed negligible for our sampler, which has a lower sampling flow rate than the Biosampler^®^, exerting lower shear stress on the collection medium. However, further investigations are required to validate this assumption.

### 4.4. qPCR tests

**Figure 5.**
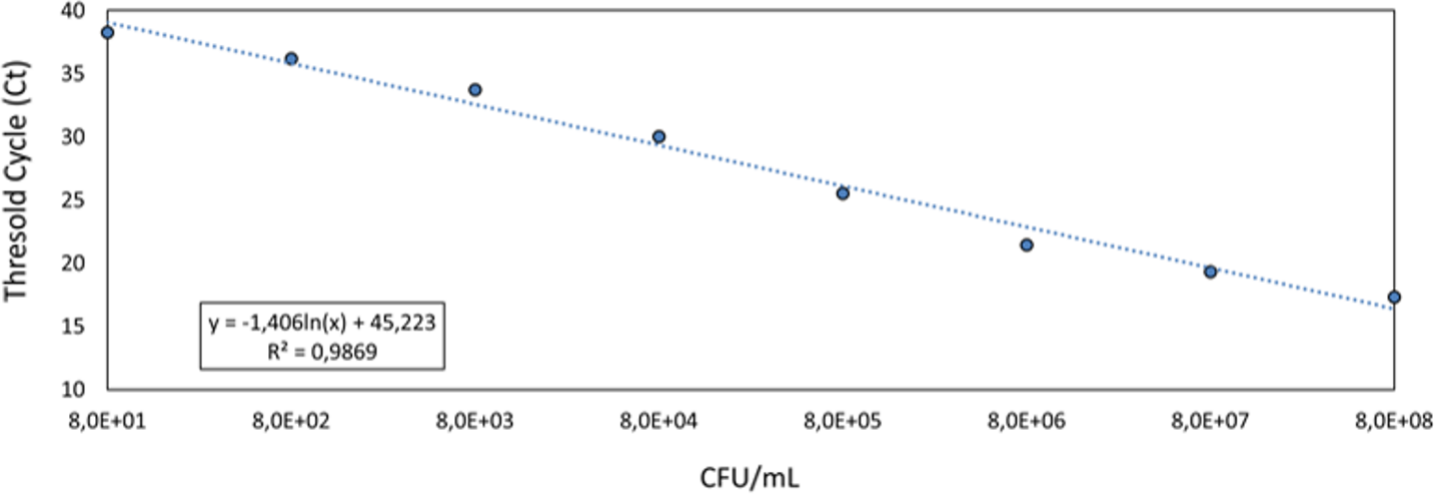
Scatter plot with correlation line between CFU ml^−1^ and corresponding qPCR Ct cycles of the calibration run performed with bacterial culture serial dilutions. The slope–intercept form and *R*-squared value are visible in the graph’s lower left corner.

The calibration run with known reference samples (*n* = 8) revealed a robust correlation (*R*-squared = 0.987) between CFU ml^−1^ and qPCR Ct values, as shown in figure 5, suggesting the suitability of qPCR for bacterial quantification in the present study.

Wilcoxon signed-rank test results (*n* = 18) showed no significant differences in qPCR Ct values before and after bioaerosol sampling for both samplers, suggesting a comparable bioaerosol sampling performance (table 5). Despite the Wilcoxon test results, the effect size values obtained suggest that qPCR Ct values before the bioaerosol collection experiments tend to be higher than after the bioaerosol collection, for both bioaerosol samplers. Effect size values suggest that the bioaerosol collection experiments had a medium effect on the Ct values when our sampler was used, and a large effect when the Biosampler^®^ was used. Lower qPCR Ct values after the bioaerosol collection experiments suggest some degree of bacterial loss in the samples. These results are in line with the bacterial culture plate count results obtained in the bioaerosol sampling tests, and can possibly be attributed to inherent and latent internal losses, as previously described by Han & Mainelis [41].

**Table 5.** Wilcoxon signed-rank test results and effect size calculated for qPCR Ct values before and after bioaerosol sampling using both bioaerosol samplers.

aerosol sampler	SOLARIS project	Biosampler^®^
significance level	0.05	
asymptotic sig.	0.553	0.086
effect size	−0.198	−0.573

A Spearman’s rank-order correlation was conducted to determine the relationship between qPCR Ct values obtained from samples collected using our sampler and the SKC Biosampler^®^. There was a strong positive correlation between the two sets of Ct values, which was statistically significant (rs = 0.776, *N* = [24], *p* = 0.03). This means that as the Ct values obtained with one sampler increase, the Ct values obtained with the other sampler tend to increase as well, further suggesting a comparable performance between the SOLARIS project sampler and the SKC Biosampler^®^.

Overall, the findings highlight the suitability of samples collected using our sampler for downstream molecular analysis using qPCR. Despite the sample size limitation, results suggest the bioaerosol collection process with our sampler did not significantly affect sample viability.

### 4.5. Scanning electron microscopy

**Figure 6.**
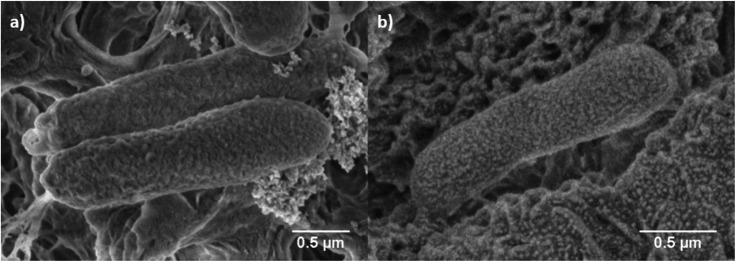
*Escherichia.coli* ATCC 25922 SEM images at 10 000× magnification. (*a*) *E. coli* samples from bioaerosol collection using the SOLARIS project sampler. (*b*) *E. coli* control samples taken from PCA cultures.

Overall bacterial morphology was well preserved in *E. coli* samples from bioaerosol collection using the SOLARIS project sampler, showing regular cell size and shape (figure 6). Occasional cellular debris was observed in both experimental samples and the *E. coli* control samples taken from PCA cultures. While cell viability could not be assessed, results suggest that bioaerosol collection using our sampler did not cause significant bacterial stress, aligning with aerosolization and qPCR results. However, further studies are necessary to investigate these findings. All in all, we can conclude from the results that samples collected with our sampler are suitable for SEM processing and imaging.

### 4.6. Aerosol sampling field trial

All samples collected with the Biosampler^®^ were negative for *C. difficile*, while non-enriched samples collected at units 2B and 3C were positive for *Clostridium perfringens*. Regarding the SOLARIS project sampler, non-enriched samples from units 2B and 3C were negative for *C. difficile* and positive for *C. perfringens*, with less than 5 CFU each. *Clostridium perfringens* spores were more concentrated in the samples than *C. difficile*, suggesting a higher concentration of *C. perfringens* spores aerosolized within the maternity units, potentially due to a higher animal colonization by these bacteria [22]. However, a higher concentration of aerosolized spores might be correlated with higher aerial dispersion, which is influenced by animal activity, airflow changes inside the facility as well as spore characteristics, among other factors. Therefore, results should be interpreted accordingly.

In the enriched air samples, the 2B unit showed no bacterial growth, while one sample from the 3C unit was positive for *C. difficile*. The overall *C. difficile* positivity rate among the swine population was 50% (25/50). Prevalence rates differed between the two maternity units, with 30.8% (8/26) in unit 2B and 70.8% (17/24) in unit 3C. All *C. difficile* isolates from both sources (animal and air) shared a common antimicrobial susceptibility profile, toxigenic profile and all belonged to the same ribotype. Results are summarized in table 6.

**Table 6.** Prevalence and characteristics of *C. difficile* isolated from bioaerosol samples collected using the SOLARIS project sampler and animal samples at the swine maternity.

unit	sample type	*C. difficile* positivity rate (*n*/*N*)	*C. difficile* ribotype
2B	non-enriched samples	0% (0/1)	NA
	enriched samples	0% (0/2)	NA
	animal samples	30.8% (8/26)	RT033
3C	non-enriched samples	0% (0/1)	NA
	enriched samples	50% (1/2)	RT033
	animal samples	70.8% (17/24)	RT033

NA, not available.

*Clostridium difficile* prevalence rate age-related differences in the animal population in the maternity units align with previous studies, indicating a correlation between intestinal microbiome immaturity and *C. difficile* overgrowth and isolation[43]. The higher colonization rate of younger animals in room 3C, along with a dynamic and densely populated environment, supports bioaerosol particle dissemination.

All *C. difficile* isolates from animals and the environment exhibit the same phenotypic and genotypic profile as the predominant RT033 clone previously described in the swine production unit [34]. These findings support a well-established transmission network involving the swine population and the production unit environment, highlighting the potential for airborne dissemination of spores contributing to the pathogen’s environmental spread. This field trial reaffirms our sampler’s utility for bioaerosol sampling and compatibility with downstream molecular analysis methods. It also emphasizes the significance of incorporating bioaerosol samples in research studies within the One Health approach.

### 4.7. Study limitations and future prospects

The CFD simulations provided valuable insights into flow patterns and particle trajectories, offering a solid foundation for understanding the sampler’s performance. While the simulations were based on specific flow parameters and modelled biological entities as 1 μm spherical particles, this approach allowed for a focused analysis that can be built upon in future studies.

Our method for estimating bacterial loss by deposition on the aerosolization chamber’s internal surface represents a novel approach. As Han and Mainelis have noted, actual bacterial losses may be higher due to various factors, including interactions between bacteria and PMMA surfaces, complex aerosol dispersion dynamics and the sample size. These findings open avenues for further research to develop a more comprehensive understanding of bacterial loss by deposition.

We assessed bacterial viability after bioaerosol sampling using culturing and SEM techniques, which yielded promising results suggesting comparable bioefficiency between our sampler and the Biosampler^®^. Future studies could incorporate additional techniques such as dye exclusion assays and nucleic acid-based methods, including propidium monoazide and ethidium monoazide coupled with qPCR (PMA/EMA-qPCR) as described by Trinh & Lee [32]. These methods could provide more accurate assessment of bacterial viability, including the detection of viable but non-culturable bacteria.

The SOLARIS project sampler demonstrated the successful performance in the field trial using culture-dependent methods. While DNA sequencing and metagenomic analysis of microbial communities were beyond the scope of this study, this presents interesting possibilities for future research.

Our study, while based on a relatively small sample size, employed rigorous methodologies to ensure the reliability of our findings. We used meticulous sampling methods, collected comprehensive qualitative data, triangulated information from multiple sources and applied statistical analyses tailored for small samples. This approach has yielded valuable initial insights into the potential of the SOLARIS project sampler for environmental bacterial collection. We view this study as an important stepping stone, laying the groundwork for more extensive research. We anticipate future studies that can build upon our findings, potentially involving larger, more diverse samples. Such research could further validate our sampler’s efficacy across a broader range of conditions and bacterial species, highlighting its potential for environmental monitoring and public health applications.

## 5. Conclusion

In this research, we successfully developed and tested the SOLARIS project sampler, a novel 3D-printed bioaerosol collection device, through a combination of CFD simulations and experimental trials. The SOLARIS project sampler, utilizing both cyclonic and impactor collection mechanisms, demonstrated efficient separation and collection of bioaerosol particles, confirming its potential as a cost-effective and accessible tool for pathogen surveillance. Additionally, a new methodology for bacterial aerosolization utilizing a custom-fabricated aerosolization chamber was established, together with a bacterial deposition method for loss assessment, which provided valuable insights for further studies in this area.

The SOLARIS project sampler demonstrated successful separation of viable *E. coli* bacteria from artificially generated bioaerosols and sample compatibility with downstream analytical methods, including microbial culturing, mass spectrometry, nucleic acid extraction for molecular detection and SEM. Moreover, comparative testing revealed that the SOLARIS sampler’s collection efficiency and bioefficiency are on par with the established SKC Biosampler^®^ under similar conditions, thereby affirming the reliability and robustness of the new device.

The field trial further underscored the practical applicability of the SOLARIS project sampler. Testing in a swine production facility successfully detected airborne pathogens such as *C. difficile* and *C. perfringens*, illustrating the sampler’s role in monitoring pathogen transmission in animal production environments. These findings emphasize the potential for airborne dissemination of pathogens and the utility of the SOLARIS sampler in contributing to One Health initiatives.

Exposure to the particulate matter (PM) fractions of air pollution, which include inhalable particles with diameters ≤10 μm (PM10), fine inhalable particles with diameters ≤2.5 μm (PM2.5) and ultrafine particles with diameters <0.1 μm (PM0.1), has been associated with higher rates of cardiovascular and respiratory disease [44]. Although environmental air pollution collection is out of the scope of this study, during our sampler’s laboratory characterization we performed aerosol collection experiments of inert 1 μm latex particles. The preliminary results obtained suggest that the SOLARIS project aerosol sampler could potentially be used for the collection of PM, which could also be an interesting avenue of research.

Overall, the SOLARIS project sampler is a valuable tool for bioaerosol collection, with applications extending to various fields of research and environmental monitoring.

## Data Availability

Original research data are publicly available in the Dryad repository [45]. Electronic supplementary material is available online [46].
